# Effects of 1-MHz Ultrasound on Epaxial Muscle Temperature in Horses

**DOI:** 10.3389/fvets.2019.00177

**Published:** 2019-06-06

**Authors:** Henry S. Adair, David Levine

**Affiliations:** ^1^Department of Large Animal Clinical Sciences, College of Veterinary Medicine, The University of Tennessee, Knoxville, TN, United States; ^2^Department of Physical Therapy, The University of Tennessee at Chattanooga, Chattanooga, TN, United States

**Keywords:** equine, ultrasound, muscle, heating, ultrasonic therapy, temperature, horse

## Abstract

**Objective:** The purpose of this study was to examine the tissue temperature changes that occur at various depths during 1.0-MHz ultrasound (US) treatments of the epaxial muscles in horses.

**Animals:** Ten healthy adult mares with no lameness or orthopedic disease weighing between 465 and 576 kg were studied.

**Procedures:** Two 1.0 MHz US treatments, one at an intensity of 1.0 W/cm^2^ and one at 2.0 W/cm^2^, were administered to the epaxial region. Needle thermistors were inserted in the epaxial muscles below the skin surface at depths of 1.0, 3.0, and 5.0 cm, directly under the US treatment area. Depths were verified with diagnostic ultrasound. Both intensities of US treatment were performed on each horse over a 20 cm^2^ area for 10 min using a sound head with an effective radiating area of 10 cm^2^. Treatments were administered in random order. Tissue temperature was measured before, during, and for an additional 10 min after the end of US treatment. Mean temperatures for each time point, location, and intensity was recorded at 30 s intervals. A mixed model analysis of variance (ANOVA) with repeated measures was used to test for differences in these means. Individual differences in the means was tested for by a Least Significant Difference (LSD) mean separation test.

**Results:** At the completion of the 10 min US treatment, the temperature rise at an intensity of 1.0 W/cm^2^ was 1.55°C at the 1.0 cm depth, 1.18°C at 3.0 cm depth, and 1.29°C at 5.0 cm depth. At an intensity of 2.0 W/cm^2^, temperatures rose 2.48°C at the 1.0 cm depth, 1.24°C at 3.0 cm depth, and 1.95°C at 5.0 cm depth.

**Conclusion and Clinical Importance:** The main findings of the study is that use of therapeutic ultrasound with a 1.0 MHz US for 10 min in horse's epaxial muscles when clipped creates the greatest heat at 1.0 cm. The heat in the tissues at 5 cm depth is more than at 3 cm depth.

## Introduction

Therapeutic ultrasound is a commonly used treatment for its thermal and non-thermal effects in treating a variety of conditions in both animals and humans (1–4). Thermal effects of ultrasound have been shown to reduce pain (5–9), decrease sub-acute and chronic edema (10–12), reduce muscle spasms (7, 13, 14), and facilitate the stretching of collagenous tissue (15–17). Non-thermal ultrasound has been shown to stimulate tissue repair, reduce pain caused by trigger points, and reduce edema (10–12). To achieve thermal benefits of ultrasound and increase tissue extensibility, tissue temperatures must be increased above normal levels (15, 16). Increasing tissue temperatures by 1°C is known to increase the tissues metabolic rate, and >2°C to reduce chronic inflammation, decrease pain, increase blood flow, decrease muscle spasms, and increase extensibility of collagen (15–21). These studies suggest that a 2–4°C increase in tissue temperature is effective in improving flexibility/range of motion (ROM) in both animals and humans (15–17). The majority of ultrasound research has been done on humans and dogs. However, therapeutic ultrasound is a modality that is often used in the equine population. Due to the lack of studies validating the effects of ultrasound on horses, further research must be conducted to establish ultrasound protocols for the equine population.

While ultrasound can be administered at many different frequencies, the majority of research has been done using 1.0 and 3.0-MHz. In human and canine studies, 1.0-MHz has been shown to heat tissue most effectively at depths between 2.0 and 5.0 cm, with 3.0 cm being the depth where maximum heating occurs (17–20, 22). Conversely, 3.3-MHz has been shown to be most effective at heating tissues between 1.0 and 2.5 cm (23, 24). In a human study, continuous 3.0-MHz ultrasound at an intensity of 0.5 W/cm^2^ was applied over the gastrocnemius muscle belly for 10 min (21). The peak increase in tissue temperature at a depth of 2.0 cm was 2.8°C (21). Additionally, the only published study evaluating the effects of therapeutic ultrasound on equine muscle and tendon temperature used a frequency of 3.3-MHz and an intensity of 1.5 W/cm^2^ (25). Using this level of ultrasound for 20 min, over a 15.0 cm^2^ area, mean temperature increase at 1.0 cm was 1.3°C, 0.7°C at 4.0 cm and 0.7°C at 8.0 cm (25).

The objective of this study was to identify the heating effect 1.0-MHz ultrasound will have at 1.0, 3.0, and 5.0 cm depths in equine epaxial muscle using intensities of 1.0 and 2.0 W/cm^2^.

## Materials and Methods

Ten adult mixed breed mares free from any orthopedic disease or lameness, weighing 465–576 kg were studied. Ages ranged from 4 to 12 years. All experimental procedures were approved by the University of Tennessee's (Knoxville, TN USA) Animal Care and Use Committee. For thermocouple placement, horses were restrained in stocks. Detomidine HCl^1^ (0.01 mg/kg intravenously {IV}) was given prior to thermocouple placement to prevent horse movement and ensure accurate placement of thermocouples. Detomidine was not administered during treatment.

Hair was clipped from a 25 cm^2^ square treatment area over the right and left longissimus dorsi muscle at the level of the second lumbar vertebrae. The center of the clipped area was 10 cm lateral to the midline. The skin in this area was prepared for aseptic insertion of sterile, hypodermic needles using povidone-iodine scrub and sterile, isotonic saline (0.9% NaCl) solution. Three mls of mepivacine HCl (20 mg/ml) was injected subcutaneously through a 25-gauge, 1.6 cm needle within a 2 cm^2^ area that was in the center of the clipped area. Within this 2 cm^2^ area, one 20-gauge, 3.81 cm needle and two, 20-gauge, 8.89 cm spinal needles were inserted. The needles were inserted toward midline at a 75° angle using diagnostic ultrasound^2^ to guarantee proper depth of 1.0, 3.0, and 5.0 cm and to avoid the lumbar vertebrae transverse process. After calibration^3^, a flexible, implantable thermistor probe^4^ was inserted through the lumen of each needle into the muscle, and the needles were removed by sliding them over the thermistor and out of the tissue. Once the needles were removed the thermistors were secured in placed by 1-inch medical tape outside the treatment area. The thermistors were connected to a microcomputer interfacing with the computer program^5^ that recorded temperature every 30 s.

**Figure 1:**
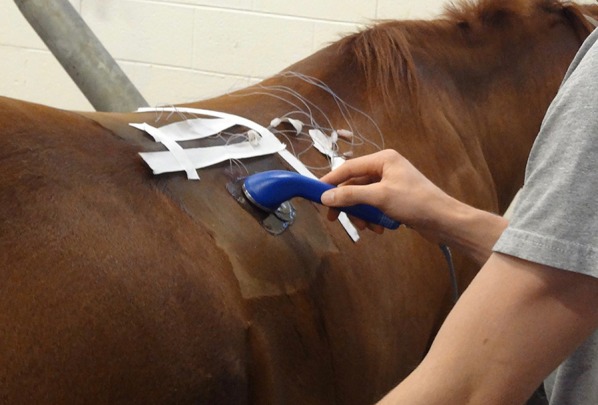
Performing the ultrasound using standard ultrasound transmission gel, for 10 min via a 1.0 MHz sound head with an effective radiating area of 10 cm^2^.

Temperature was recorded for 5 min to establish baseline temperature at each treatment depth. A 20 cm^2^ template was used to outline the treatment area centered at the thermistor insertion area. Ultrasound was applied, using standard ultrasound transmission gel, for 10 min via a sound head with an effective radiating area of 10 cm^2^. The ultrasound unit^6^ was calibrated^7^ by an authorized calibration service immediately prior to use in this study. The 20 cm^2^ area received 10 min of continuous ultrasound at 1-MHz and either 1.0 or 2.0 W/cm^2^ that was determined through random assignment (either right or left longissimus muscle) (Figure 1). Temperature was recorded every 30 s during treatment and for 10 min after treatment ended. At the end of the measurement period, the thermistors were removed and each horse was administered phenylbutazone (4.4 mg/kg IV).

### Statistical Analysis

Mean temperatures for each time point, location, and intensity were calculated using computer software^8^. A mixed model analysis of variance (ANOVA) with repeated measures was used to test for differences between the heating obtained at the three different depths, and for the two different intensities at these three depths. Individual differences in the means was tested for by a Least Significant Difference (LSD) mean separation test. To test if tissue temperature change over time at each location and depth was significantly different, area under the curve (AUC) was calculated with a log linear trapezoid rule. ANOVA and LSD mean separation were calculated by computer software^9^. Results were considered significant at *P* < 0.05.

## Results

Implantation of thermistors and ultrasound treatment were well-tolerated by all horses. At the site of thermistor implantation the longissimus dorsi muscle averaged 8 cm in depth (range 6–9 cm). No post-procedure complications were encountered.

**Figure 2:**
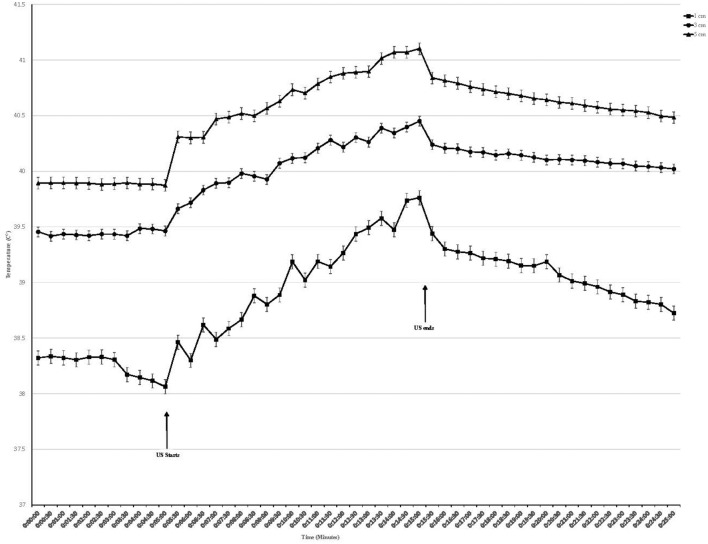
Tissue temperature change in epaxial muscles during therapeutic ultrasound at 1.0 W/cm^2^. Ultrasound starts at 5 min and ends at 15 min. Values presented as mean ± SEM.

**Figure 3:**
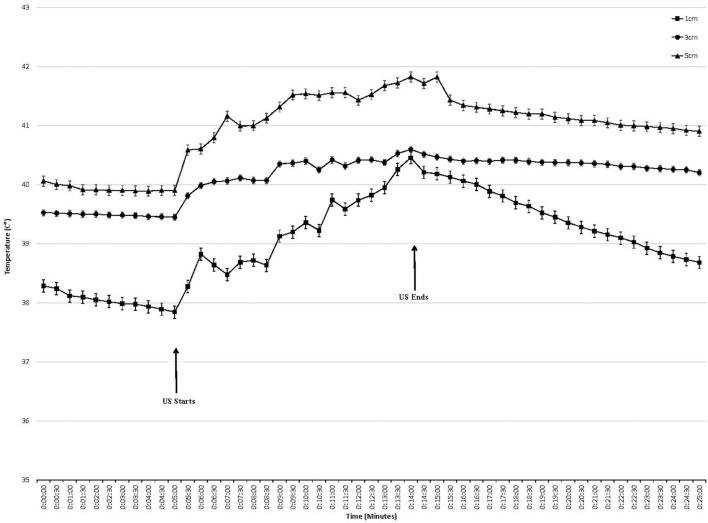
Tissue temperature change in epaxial muscles during therapeutic ultrasound 2.0 W/cm^2^. Ultrasound starts at 5 min and ends at 15 min. Values presented as mean ± SEM.

Temperature began to rise within 30 s of initiating treatment at both intensities and at all depths (Figures 2, 3). At the termination of treatment there was a slow but steady decline in temperature at both intensities and at all depths. However, at the end of the measurement period (10 min after treatment ended) temperatures at both intensities and all depths were still above baseline. Baseline temperature was 38.3, 39.4, and 39.8°C at a depth of 1.0, 3.0, and 5.0 cm, respectively.

When using an intensity of 1.0 W/cm^2^, the maximum rise in temperature in the epaxial musculature at a depth 1.0 cm was 1.55°C, and this temperature was reached at the end of treatment. At a depth of 3 cm, the maximum rise in temperature was 1.18°C, which was reached at the end of treatment. At a depth of 5 cm, the maximum rise in temperature was 1.29°C, which was reached at the end of treatment (Table 1).

**Table 1:** Increase in tissue temperature after 10 min treatment time with a therapeutic ultrasound.

**Tissue depth**	**Baseline temperature**	**Conclusion of 10 min US (peak temperature)**	**Ten minutes following treatment end**
**1.0 W/cm**^**2**^ **ULTRASOUND INTENSITY**			
1.0 cm	38.3°C, 95% CI [38.1–38.5]	39.8°C, 95% CI [39.7–39.9]	38.7°C, 95% CI [38.6–38.8]
3.0 cm	39.4°C, 95% CI [39.2–39.6]	40.5°C, 95% CI [40.4–40.6]	40.0°C, 95% CI [39.9–40.1]
5.0 cm	39.8°C, 95% CI [39.6–40.0]	41.1°C, 95% CI [41.0–41.2]	40.5°C, 95% CI [40.4–40.6]
**2.0 W/cm**^**2**^ **ULTRASOUND INTENSITY**			
1.0 cm	38.1°C, 95% CI [37.9–38.3]	40.5°C, 95% CI [40.4–40.6]^*^	38.7°C, 95% CI [38.6–38.5]
3.0 cm	39.4°C, 95% CI [39.2–39.6]	40.6°C. 95% CI [40.4–40.8]^*^	40.2°C, 95% CI [40.0–40.4]
5.0 cm	39.8°C, 95% CI [39.6–40.0]	41.8°C, 95% CI [41.7–41.9]^*^	40.9°C, 95% CI [40.8–41.0]

Values denoted by

* *significantly different from 1.0 W/cm^2^ (P < 0.05)*.

When using an intensity of 2.0 W/cm^2^, the maximum rise in temperature in the epaxial musculature at a depth 1.0 cm was 2.48°C, and this temperature was reached 9 min into treatment. At a depth of 3 cm, the maximum rise in temperature was 1.24°C, which was reached 9 min into treatment. At a depth of 5 cm, the maximum rise in temperature was 1.95°C, which was reached 9 min into treatment (Table 1).

At a depth of 1.0, 3.0, and 5.0 cm, the 2.0 W/cm^2^ intensity heated tissues significantly more than the 1.0 W/cm^2^ intensity (*p* < 0.01) (Table 1).

Ten minutes after the conclusion of the treatment, the tissues temperatures for the 1.0 W/cm^2^ setting were still elevated from baseline with temperatures of 38.7, 40.0, and 40.5°C for depths of 1.0, 3.0, and 5.0 cm, respectively. For the 2.0 W/cm^2^ the tissue temperatures were 38.7, 40.2, and 40.9°C for depths of 1.0, 3.0, and 5.0 cm, respectively.

## Discussion

Tissue temperatures at baseline were greatest at the 5.0 cm depth, followed by 3.0 cm, followed by the 1.0 cm depth. This was anticipated, as the deeper tissues are closer to the body's core and consistent with previous studies on horses, dogs, and humans (18, 19, 22–24) (Figures 2, 3).

While statistical significance was reached between 2.0 and 1.0 W/cm^2^ intensities at all depths, the difference at 3.0 cm was small and likely not clinically significant. Statistical significance was reached in the 3.0 cm condition as the values all trended upward (though just slightly) when comparing 2.0–1.0 W/cm^2^.

The difference in the overall increase between the 3 cm depth vs. the 5 cm depth was unexpected. If one considers tissue absorption and the half-value layer (the depth by which 50% of the ultrasound beam intensity is absorbed into the tissue, reducing the intensity as it travels through tissue) one would predict that the temperature at 5 cm would be lower than the temperature at 3 cm (26). However, Demmink et al., utilizing cadaver tissues, found that different tissue geometries (different thermal and acoustical properties can influence the depth limit for the different temperature ranges (27). Utilizing thermal images that only depicted the tissue temperature change, they demonstrated that tissue geometry and properties have an influence on the heating depths (27). Ultrasound penetrates through tissue high in water content and is absorbed in dense tissues that are high in protein where it will have its greatest effects (23). The denser the medium, the more the ultrasound beam will be absorbed by the tissues and possibly result in a greater heating effect (23). Highly collagenous regions that may be exposed include superficial cortical bone, periosteum, menisci, synovium and capsules of joints, myofascial interfaces, intermuscular scars, fibrotic muscle, tendon sheaths, and major nerve trunks (28). If the 5 cm thermocouple was closer to the bone/muscle interface then a higher temperature could occur due to reflection off of the bone. In this study we used diagnostic ultrasound to determine location of the thermistor tip and to try and ensure that the thermistors were placed only in muscle. The average depth of longissimus dorsi muscle at this level was 8 cm before the transverse process was encountered. Since the deepest thermistor was at 5 cm the possibility heating from the bone was low. However, the longissimus dorsi muscle has fascial planes that may absorb more of the ultrasound beam thus heating more that the underlying muscle. We made no attempt to avoid these fascial planes so excess heat generated by the increased collagenous tissue may have occurred. Additionally, one must consider the thermoregulation effect of blood circulation (27). The muscle tissue at the 3 cm depth may have had higher circulation leading to a greater rate of heat dissipation.

The effects of detomidine sedation must be considered when performing ultrasound treatment. One must avoid profound sedation so that the horse is able to avoid excessive heat in the tissues. The dose of detomidine used in this study has been shown not to effect the ability of the horse to detect and avoid a noxious thermal stimulus (29, 30). The effects of detomidine administration on muscle function must also be considered. Edner et al. studied the relationship of muscle perfusion and metabolism with cardiovascular variables before and after detomidine injection during propofol/ketamine anesthesia in horses (31). They found that detomidine caused profound hypertension and bradycardia and decreased cardiac output and muscle perfusion (31). However, 10 min after detomidine injection muscle perfusion had recovered to pre-injection levels (31). Since measurements were not begun until 30 min after detomidine administration the effects on muscle perfusion should be minimal. Kruljc and Nemec found that a dose of 0.022 mg/kg significantly reduced muscle EMG activity (32). However, this dose was twice that used in this study. Wooldridge et al, found no effect of detomidine on esophageal skeletal muscle and postulated that the previously reported effect may be centrally mediated (33). Since this study was evaluating the thermal effect of ultrasound on muscle, a centrally mediated effect on muscle function should not affect the results.

Previous studies suggest that a 2–4°C increase in tissue temperature is effective in improving flexibility/range of motion (ROM) in both animals and humans (15–17). The use of 1.0-MHz US in this study caused >2.0°C tissue-temperature elevation in only 1 of the 6 US conditions (2.0 W/cm^2^, 1.0 cm depth). Two other conditions were close to reaching a 2°C increase (1.0 W/cm^2^ at 1 cm depth and 2.0 W/cm^2^ at the 5 cm depth). Increasing either the treatment time or the US intensity may have achieved an increase in tissue temperature to therapeutic levels in these conditions. In comparing intensities, the 2.0 W/cm^2^ intensity elevated tissue temperature significantly higher than the 1.0 W/cm^2^ intensity at all depths. Because the temperature increase was short-lived in all conditions studied, for optimal gains in range of motion, stretching should be applied if possible, during the last half of the treatment, and immediately after cessation of treatment.

## Conclusions

This study demonstrates that statistically significant heating occurs in the epaxial muscles of horses during 1.0 MHz US with the greatest heating occurring at a depth of 1.0 cm. However, the lower end of the therapeutic range of tissue heating was only reached at 2.0 W/cm^2^, 1.0-cm depth. Increasing treatment time or US intensity may lead to further increase in tissue temperature.

## Ethics Statement

All experimental procedures were approved by the University of Tennessee's (Knoxville, TN, USA) Animal Care and Use Committee.

## Author Contributions

HA and DL were involved in study design, data collection, data interpretation, and preparation of the manuscript.

### Conflict of Interest Statement

The authors declare that the research was conducted in the absence of any commercial or financial relationships that could be construed as a potential conflict of interest.

## Footnotes

^1^Dormosedan®: Zoetis United States, Florham Park, NJ, USA.

^2^MyLab 40, Esaote, Indianapolis, IN.

^3^InstaCal 5.83, Measurement Computing Corporation, Norton, MA.

^4^Thermes USB, Physitemp Instrument, Inc., Clifton, NJ.

^5^DasyLab 9, Measurement Computing, Norton, MA.

^6^Mettler Sonicator 730x, Mettler Electronics Corp, Anaheim, CA.

^7^TTT Medical Equipment Repair, 2372 Salem Valley Road. Ringgold, GA.

^8^Microsoft Excel 2016, Microsoft, Redmond, WA.

^9^SAS Institute, Cary, NC.
